# Twenty-Four-Hour Urine Osmolality as a Physiological Index of Adequate Water Intake

**DOI:** 10.1155/2015/231063

**Published:** 2015-03-18

**Authors:** Erica T. Perrier, Inmaculada Buendia-Jimenez, Mariacristina Vecchio, Lawrence E. Armstrong, Ivan Tack, Alexis Klein

**Affiliations:** ^1^Danone Research, 91767 Palaiseau, France; ^2^Department of Kinesiology, Human Performance Laboratory, University of Connecticut, Storrs, CT 06269, USA; ^3^Service des Explorations Fonctionnelles Physiologiques et INSERM 1048, Equipe 12, CHU de Rangueil, 31432 Toulouse, France

## Abstract

While associations exist between water, hydration, and disease risk, research quantifying the dose-response effect of water on health is limited. Thus, the water intake necessary to maintain optimal hydration from a physiological and health standpoint remains unclear. The aim of this analysis was to derive a 24 h urine osmolality (U_Osm_) threshold that would provide an index of “optimal hydration,” sufficient to compensate water losses and also be biologically significant relative to the risk of disease. Ninety-five adults (31.5 ± 4.3 years, 23.2 ± 2.7 kg·m^−2^) collected 24 h urine, provided morning blood samples, and completed food and fluid intake diaries over 3 consecutive weekdays. A U_Osm_ threshold was derived using 3 approaches, taking into account European dietary reference values for water; total fluid intake, and urine volumes associated with reduced risk for lithiasis and chronic kidney disease and plasma vasopressin concentration. The aggregate of these approaches suggest that a 24 h urine osmolality ≤500 mOsm·kg^−1^ may be a simple indicator of optimal hydration, representing a total daily fluid intake adequate to compensate for daily losses, ensure urinary output sufficient to reduce the risk of urolithiasis and renal function decline, and avoid elevated plasma vasopressin concentrations mediating the increased antidiuretic effort.

## 1. Introduction

Hydration is a dynamic balance between water intake and loss, and body water balance is maintained through both behavioral and physiological responses. Water gains come almost entirely from fluids and water in food, as metabolic water accounts for only a small fraction of daily water gain. Adequate intakes representing in fact population median consumption have been reported [1, 2]; however, the intake necessary to maintain optimal hydration from a physiological and health standpoint is still unknown. This is in part because although water is essential to sustain life and normal physiological functions, research quantifying the dose-response effect of water on health is very limited. Relationships between water and health outcomes are difficult to assess. Body water needs are highly individual and depend upon body composition and body size, diet-related osmotic load, physical activity and fitness level, and other factors such as climate, environment, and disease. Moreover, accurately measuring water intake is difficult, especially over long periods of time, and relatively little research has focused on average adults with mostly sedentary lifestyles and occupations [3].

Despite the challenges to linking water intake to health, the available evidence suggests that insufficient water intake is linked to some long-term health issues. Specifically, recent studies linking water intake and disease suggest a relationship between water, hydration, and recurrent kidney stone disease (urolithiasis) [4–6], chronic kidney disease [7–9], blood glucose regulation [10], and cardiovascular events [11]. Collectively, these observations provide preliminary evidence that daily water intake* above the minimum which is physiologically necessary to maintain total body water* may confer additional benefits above simply maintaining total body water. It can therefore be argued that truly adequate water intake is one which replaces daily losses, maintains total body water, and provides relative risk reduction for the development of various long-term pathologies linked to low water intake. However, considering large interindividual differences in water needs, there is a need for a quantitative measurement, a physiological index or biomarker that would allow individuals to assess the adequacy of their daily water intake.

The physiological regulation of water balance is dynamic and complex, and biomarkers in plasma and urine have been commonly used to assess hydration. Plasma osmolality is a biomarker sensitive to acute dehydration [12, 13], but it is maintained within a narrow range across a broad spectrum of daily fluid intake volumes [2, 14]. Small increases in plasma osmolality, sensed by osmoreceptors located primarily in the brain, trigger a release of the hormone arginine vasopressin (AVP) from the pituitary gland. The increase in plasma AVP increases water reabsorption in the kidney, reducing the volume of water lost via urine, increasing urine concentration, and protecting plasma osmolality. Thus, the measurement of plasma osmolality represents the* outcome* (i.e., the successful maintenance of total body water and plasma osmolality), but not the* process* (i.e., antidiuresis via the urine concentrating mechanism) involved in maintaining body water balance.

It is precisely this mechanism, the continuous adjustment of urine flow to maintain plasma osmolality, which allows biomarkers of urine concentration to be responsive to differences in fluid intake volume [14–18]. Urinary output and, more specifically, urine concentration are the end result reflecting the antidiuretic activity required to maintain water balance in response to varying levels of water intake and loss. Armstrong and colleagues argue that 24 hour urine osmolality (U_Osm_) is an excellent indicator of 24 h hydration because it “represents the sum of all behavioral and neuroendocrine responses which influence renal concentration or dilution throughout a day” (article under review). It can therefore be argued that 24 h U_Osm_ is the biomarker most suitable to determine appropriate fluid intake* for the individual*, because it reflects the net sum of water gains, losses, and neuroendocrine regulatory responses. This urinary variable integrates differences in body size, physical activity, and dietary solute load that are difficult to normalize in population-based recommendations. Thus, it seems plausible that a desirable 24 h U_Osm_ threshold may be established. The aim of this analysis was to derive a 24 h U_Osm_ threshold that represents “optimal hydration”—that is, a daily fluid intake sufficient to compensate all losses and maintain a urinary output which may help to reduce the risk of chronic disease.

## 2. Methods

### 2.1. Participants and Procedure

The study was approved by the appropriate ethics committee (Comité de Protection des Personnes CPP XI in Saint-Germain-en-Laye, France) and all participants provided written informed consent. Ninety-five healthy, community-dwelling French adults (age: 31.5 ± 4.3 years, BMI: 23.2 ± 2.7 kg·m^−2^, 52% females) collected 24 h urine samples, provided fasting (morning) blood samples, and completed food and fluid intake diaries over 3 consecutive weekdays. Participants collected 3 consecutive 24 hour urine samples, which were checked for completeness [14]. Urine samples were weighed on a precision scale, osmolality was analyzed using the freezing point depression method (Messtechnik, Inc.), and specific gravity (U_sg_) was measured using a hand-held digital refractometer (PEN-Urine SG, ATAGO Inc.). Urine volume (U_Vol_) was calculated to the nearest mL from urine mass and U_sg_. Plasma AVP (P_AVP_) concentration was assessed from morning blood samples. All food and fluid consumption was recorded using a custom online food and fluid e-diary (Neometis-24WQ-Waters; MXS, France). Details of the study protocol, food and fluid records, urine collections, and biochemical analyses have been described elsewhere [14].

### 2.2. Analytical Approach

To date, the adequacy of water intake has been evaluated relative to dietary guidelines, or disease risk, or biomarkers of hydration. This analysis attempted to reconcile these three approaches into a combined, quantitative biomarker representing adequate intake for optimal hydration. To determine a threshold for 24 h U_Osm_ representing adequate intake, three strategies were used. U_Osm_ was assessed relative to dietary reference values, disease risk, and neuroendocrine control of water balance to determine whether these three approaches converged about a common, biologically-significant 24 h U_Osm_ threshold. Specifically, urine osmolality was examined in relation to (1) existing European water intake dietary reference values [1]; (2) recent publications linking fluid intake and urine volume to lithiasis [4] and chronic kidney disease [7]; and (3) P_AVP_ concentration. Statistical methods varied as a function of each approach, and included the use of ANOVA and receiver-operating characteristic (ROC) analysis to determine optimal U_Osm_ cut-offs. For each strategy, the statistical methodology is presented in more detail. Moreover, due to the unique analytical approach of this study, in which three strategies are evaluated sequentially in order to arrive at a common conclusion, a brief discussion relevant to each strategy will be presented directly after each analysis. A general conclusion will follow.

## 3. Results

### 3.1. Fluid Intake and Urine Output

**Figure 1:**
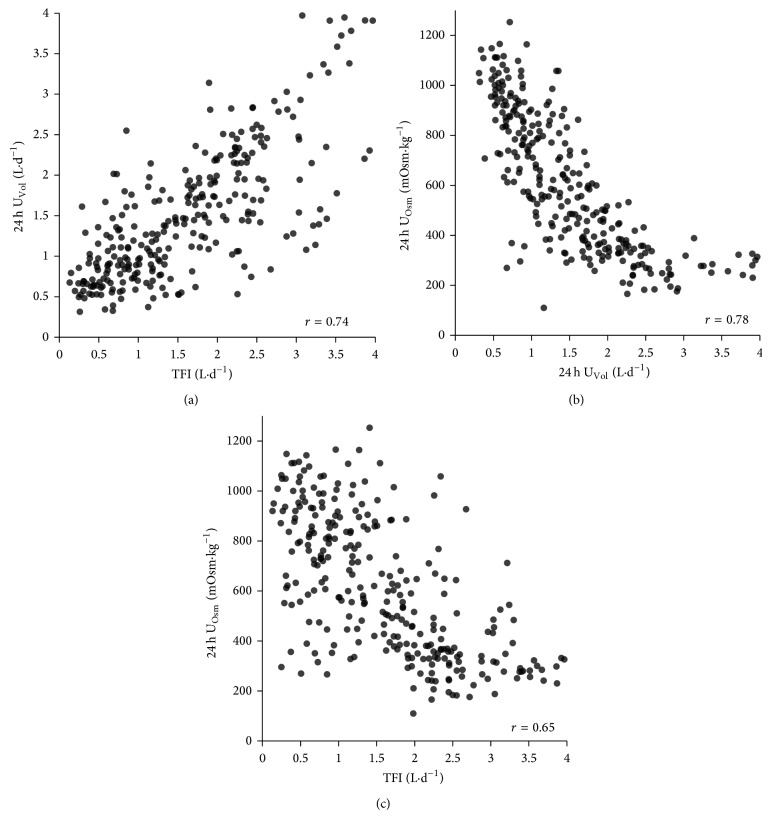
Distributions of 24 h total fluid intake, urine volume, and urine osmolality.

The mean ± SD (10th percentile to 90th percentile) for fluid intake and urine output were total daily fluid intake (TFI) 1.6 ± 0.9 L·d^−1^ (0.48 to 3.0  L·d^−1^); 24 h U_Vol_, 1.5 ± 0.8 L (0.6 to 2.5 L); 24 h U_Osm_, 609 ± 274 mOsm·kg^−1^ (277 to 999 mOsm·kg^−1^). There were moderate to strong correlations between each pair of variables (Figure 1).

### 3.2. Strategy 1: Determine the Optimal 24 h U_Osm_ Value to Distinguish Daily Fluid Intake Volume that Satisfies the Dietary Reference Intake for Water

**Figure 2:**
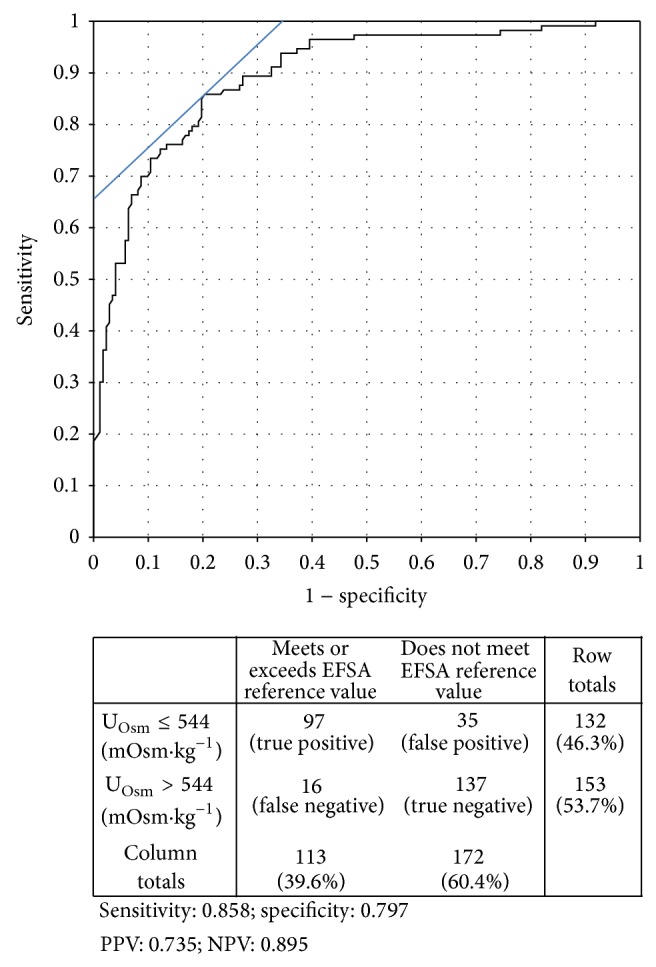
ROC analysis curve for urine osmolality as an indicator of fluid intake meeting EFSA fluid intake guidelines. AUC = 0.895; optimal cutoff 544 mOsm·kg^−1^.

European Food Safety Authority (EFSA) adequate intake for total water assumes that, on average, 70–80% of TWI is obtained through fluid consumption. Thus, the reference values for TFI can be approximated as 2.0 and 1.6 L·d^−1^ for adult men and women, respectively. In order to determine the 24 h U_Osm_ cut-off that would best discriminate between subjects who met or exceeded and those who did not meet the EFSA reference values for fluid intake, a binary variable was constructed based on participants' 24 h TFI (Yes: meets or exceeds the sex-specific reference value for intake; No: does not meet reference value). Logistic regression of 24 h U_Osm_ against this binary outcome was performed, and a ROC analysis was used to determine the optimal U_Osm_ cutoff value. No adjustment was made to favor either sensitivity or specificity. The optimal 24 h U_Osm_ for distinguishing between those who met or exceeded and those who did not meet EFSA TFI reference values was 544 mOsm·kg^−1^ (area under the curve = 0.895; Figure 2).

In establishing daily reference values for TWI, the EFSA panel relied in part on a calculation of the theoretical amount of urine required to excrete 24 h solute at a “desirable urine osmolarity” of 500 mOsm·L^−1^ [1]. The cut-off determined using the ROC analysis (544 mOsm·kg^−1^) is consistent with this EFSA calculation. The similarity between the experimentally-derived cutoff in this sample and the EFSA theoretical calculation is remarkable, considering that the EFSA calculations relied on average dietary solute ingestion from various European dietary population surveys. In our sample, 24-hour urine from European participants in normal daily living conditions, consuming their normal daily food and fluids, provides experimental support for a 24 h U_Osm_ threshold approaching 500 mOsm·kg^−1^ as a way to evaluate whether total fluid intake meets European reference values.

### 3.3. Strategy 2: Consider 24 h U_Osm_ and Relative Risk for Disorders of the Kidneys and Urinary Tract

#### 3.3.1. Urolithiasis

The relationship between fluid intake, urine volume, and kidney stones has been widely reported [11, 19–22]; however, few studies have evaluated the risk of stone formation relative to specific volumes of fluid intake or urine output. In a prospective examination of data from the Nurses' Health Study (NHS), Curhan et al. [4] determined that the multivariate-adjusted odds-ratio (OR) for kidney stones was lower when TFI exceeded 1850 mL·d^−1^ in a sample of young women. Using the female participants in our sample (*n* = 49 women providing 147 fluid records and corresponding 24 h urine collections), logistic regression and ROC analysis with a binary outcome of FI greater than or less than 1850 mL·d^−1^ showed the optimal 24 h U_Osm_ cutoff for distinguishing those who met the fluid intake threshold for reduction in stone risk to be 525 mOsm·kg^−1^ (AUC = 0.92; sensitivity 0.95; specificity 0.77). This provides a second, independent support for a 24 h hour U_Osm_ threshold approaching 500 mOsm·kg^−1^ as an indicator of adequate total fluid intake.

A threshold of 500 mOsm·kg^−1^ is also supported by data from the only prospective, randomized controlled trial of increased water intake to prevent stone recurrence [23]. Borghi et al. reported that in a population of stone-formers, increasing 24 h U_Vol_ above 2 L·d^−1^ (U_Vol_, 2.1–2.6 L) resulted in a 50% lower stone recurrence rate over 5 years. In our study sample, 79% of urine collections were accurately classified as being above or below 2 L·d^−1^ based upon a U_Osm_ cutoff of 500 mOsm·kg^−1^ (98% sensitivity, 74% specificity). Moreover, the optimal cut point for distinguishing 24 h U_Vol_ above or below 2 L·d^−1^ also approached 500 mOsm·kg^−1^ (448 mOsm·kg^−1^; 98% sensitivity, 82% specificity).

This analysis of 24 h U_Osm_ relative to stone disease risk, performed independently of the analysis relative to EFSA intake guidelines, is especially interesting considering that both methods converge on approximately 500 mOsm·kg^−1^ as a desirable 24 h U_Osm_ (Table 1). The advice to stone-formers to increase their urine volume is common in clinical practice, and several studies have confirmed the importance of high fluid intake and urine output on secondary stone prevention. The underlying principle of these recommendations is to dilute urinary solute concentration and prevent supersaturation and crystallization. While not sufficient on its own, urine dilution is a key component in the therapeutic strategy for preventing recurrence in renal stone formers. Since the goal is to prevent supersaturation by diluting urine, there is a rationale for taking into account total urine osmolyte content and concentration. Targeting a 500 mOsm·kg^−1^ urine osmolality threshold rather than a “universal” optimal urine volume (2 L/day is a common recommendation) represents an advantage: it takes into account individual metabolic constraints and provides a simple way to assess by simply checking urine color or specific gravity. In this sense, a 24 h U_Osm_ target outperforms both water intake and urine volume-based recommendations, as it is the only measure capable of accounting for differences in intake, loss, and dietary solute load, which will influence the minimum water requirement. Thus, establishing a U_Osm_ target of ≤500 mOsm·kg^−1^ as a physiological index of hydration appears relevant both for tracking adequate intake in the general population, as well as for specific patient groups such as stone formers.

#### 3.3.2. Chronic Kidney Disease

**Figure 3:**
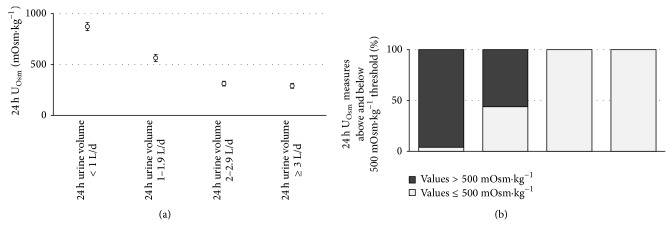
(a) Mean (95% CI) 24 h urine osmolality and (b) proportion of urine osmolality measures above (light grey) and below (dark grey) 500 mOsm·kg^−1^ according to urine volume categories reported by Clark et al. [7].

Recent studies have reported that low 24 h urine volume and low daily fluid intake are associated with a higher risk of chronic kidney disease [7, 9]. Clark and colleagues assessed the rate of annual eGFR decline in 2148 adults over 7 years and found an inverse, graded, dose-response relationship between 24 h U_Vol_ and annual renal decline. Mild to moderate renal decline was slowest in people with the highest U_Vol_ (≥3 L·d^−1^: multivariate-adjusted OR [95% CI]: 0.66 [0.46–0.94]; 2.0–2.9 L·d^−1^: OR 0.84 [0.67–1.05]) and fastest in those with lowest U_Vol_ (<1 L·d^−1^: OR 1.33 [1.01–1.75]), compared to the reference urine volume. Using the dataset described above and the 4 U_Vol_ categories described by Clark et al., ANOVA revealed that 24 h U_Osm_ was significantly different between categories of U_Vol_ (F [3,281] = 148; *P* < 0.0001). Mean [95% CI] 24 h U_Osm_ was 872 [837,906]; 564 [533,594]; 312 [265,359]; and 290 [200,380] mOsm·kg^−1^ in the respective urine volume categories (<1.0; 1.0–1.9; 2.0–2.9; ≥3.0 L/day).  Figure 3 illustrates that when 24 h urine volume was less than 1 L·d^−1^, corresponding to Clark's highest category of relative risk, 96% of U_Osm_ values were >500 mOsm·kg^−1^. In contrast, when 24 h urine volume was ≥2 L·d^−1^L, 100% of U_Osm_ values were ≤500 mOsm·kg^−1^.

Clear-cut thresholds for biological variables rarely exist, and individual differences in water metabolism are no exception. The two independent approaches described above, using intake reference values and disease risk, do not result in identical U_Osm_ cutoffs. However, the fact that all approaches converged around approximately 500 mOsm·kg^−1^ is quite remarkable. The optimal U_Osm_ cutoff to determine which adults met or exceeded intake guidelines [1, 2] was 544 mOsm·kg^−1^; the cutoff for women who met the fluid intake volume for kidney stone risk reduction [4] was 525 mOsm·kg^−1^; and the cutoff for adults who increased their urine volume for secondary stone prevention [23] was 448 mOsm·kg^−1^. Moreover, the urine volume associated with risk reduction for chronic kidney disease further supports a desirable 24 h U_Osm_ of less than 500 mOsm·kg^−1^. Thus, it appears that a 24 h U_Osm_ threshold of ≤ 500 mOsm·kg^−1^ is suggestive of a daily fluid intake that is adequate to (a) satisfy European intake reference values and (b) play a role in the prevention of pathologies of the kidneys and urinary tract.

### 3.4. Strategy 3: Assess P_AVP_ Concentration in Relation to U_Osm_

AVP is the major actor in a cascade of hormones that regulate the permeability and absorption of water in the kidneys, maintaining total body water homeostasis across a wide range of fluid intake volumes. P_AVP_ concentration is thus a direct reflection of antidiuretic effort to preserve total body water in response to insufficient intake or increased water loss. Of the 285 records of daily fluid intake and urine osmolality, 283 had associated P_AVP_ measures. Mean P_AVP_ concentration was compared across 3 U_Osm_ categories: ≤500 mOsm·kg^−1^, corresponding to the threshold for intake adequacy proposed in this paper; >800 mOsm·kg^−1^, corresponding to an indication of mild dehydration [24]; and 501–800 mOsm·kg^−1^. ANOVA revealed significant differences in P_AVP_ between the three U_Osm_ categories [F (2,720) = 34.01; *P* < 0.0001]. P_AVP_ concentration across each ascending category of U_Osm_ was (mean [95% CI]) 1.76 [1.59,1.93]; 2.39 [2.11,2.67]; and 3.2 [2.86,3.54] pg·mL^−1^, respectively, representing a mean increase of 0.6 to 0.8 pg·mL^−1^ between U_Osm_ categories.

**Figure 4:**
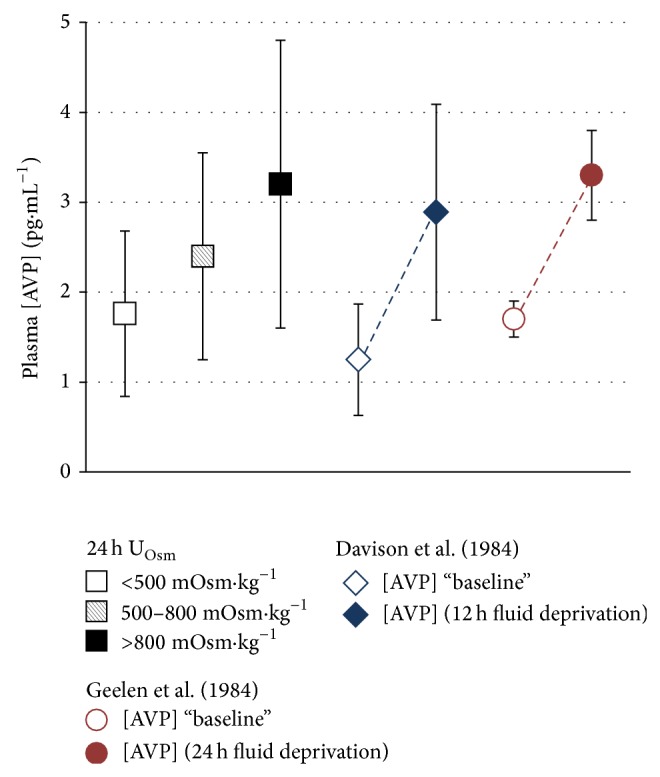
From left to right: mean (standard deviation) plasma AVP concentration grouped by 24 h U_Osm_ (<500; 500–800; >800 mOsm·kg^−1^); plasma AVP reported before and after 12 h of total fluid deprivation [25]; plasma AVP reported before and after 24 hours of total fluid deprivation [26].

While comparative data are limited, the mean P_AVP_ when U_Osm_ ≤ 500 mOsm·kg^−1^ was very similar to baseline AVP concentrations reported in previous studies (Figure 4). The mean AVP concentration when U_Osm_ was between 501 and 800 mOsm·kg^−1^ was similar to P_AVP_ previously reported in female subjects after 12 hours of fluid deprivation [25], and the mean P_AVP_ when U_Osm_ was greater than 800 mOsm·kg^−1^ was similar to values previously reported during 24 hours of fluid deprivation [26]. This reveals that the antidiuretic efforts of the kidney are increased even when 24 h urine osmolality is below 800 mOsm·kg^−1^ and suggests that even moderately high U_Osm_ (501–800 mOsm·kg^−1^) is indicative of increased antidiuretic effort to maintain total body water.

Osmotic stimulation of AVP is quite sensitive, and changes of less than 1% in plasma osmolality are capable of triggering the release of AVP. It has been reported that an increase of 1 mOsm·kg^−1^ in plasma produces a change of between 0.4 and 0.8 pg·mL^−1^ in circulating AVP [27]. Similarly, the renal response to increased circulating AVP is equally sensitive, with maximal urine concentration occurring when plasma AVP concentration approaches 4 to 5 pg·mL^−1^. While the differences in plasma AVP observed between U_Osm_ thresholds are quantitatively small, they are clinically significant in terms of their impact on the renal urine concentrating mechanism: small, initial increases from baseline levels of plasma AVP have a much greater relative effect to decrease urine flow than do further increases approaching 5 pg·mL^−1^ [27]. Thus, even without a significant reduction in total body water, insufficient fluid intake appears to mimic the hormonal profile and urine concentration seen in fluid restriction and dehydration.

## 4. Discussion

Daily water needs are highly individual due to differences including body size, climate and environment, daily activities, metabolism, and dietary solute load. However, given that water intake recommendations are constructed to satisfy the general needs of a population, it is difficult for individuals to objectively assess whether they are drinking enough to meet their specific needs. The present study aimed to determine a 24 hour U_Osm_ index which would reflect an adequate daily fluid intake for optimal hydration, using three approaches: (1) comparisons with intake recommendations from EFSA [1]; (2) associations between intake, urinary output, and diseases of the kidneys and urinary tract; and (3) associations between U_Osm_ and AVP. Together, the first and second approaches suggest that a 24 hour U_Osm_ of less than or equal to 500 mOsm·kg^−1^ (which will be referred to, for simplicity, as U500) represents an intake that is adequate to compensate for daily losses and ensure a urinary output sufficient to reduce the risk of urolithiasis and decline in renal function. Moreover, U500 was supported by the third approach, which revealed that 24 h urine osmolality of >500 mOsm·kg^−1^ was associated with elevated plasma AVP concentrations suggestive of antidiuretic effort.

Previous work to establish a U_Osm_ threshold has focused on the detection of dehydration, rather than on the adequacy of intake for health. A 24-hour U_Osm_ threshold of approximately 800 mOsm·kg^−1^ to detect dehydration has been supported both in children and adults. Manz and Wentz [28] defined 830 mOsm·kg^−1^ as the lower bound for hypohydration in children, based on a calculation of the mean maximal urine concentrating capacity, minus 2 standard deviations, thereby covering the “minimum maximal urine concentrating capacity” of 98% of the population described (i.e., European children and adolescents consuming a protein-rich western diet). A nearly identical criterion value for mild dehydration (831 mOsm·kg^−1^; 91% sensitivity, 91% specificity) was reported in a sample of adults [13]. For acute dehydration, a threshold of ~800 mOsm·kg^−1^ appears to be reasonable. However, drinking just enough to maintain a 24 h U_Osm_ below 800 mOsm·kg^−1^ may not be sufficient to ensure optimal hydration, if one considers not only dehydration but also the risk for health impact. Growing evidence suggests that a truly adequate intake requires drinking* more than strictly physiologically necessary*.

The body's ability to preserve plasma osmolality and total body water despite differences in water intake is due to the sensitive regulation of urine concentration by the kidneys, largely modulated through the release of the antidiuretic hormone, AVP. Although low water intake can be compensated by high antidiuretic activity and a low urine output, the risk for some chronic diseases appears to be associated with low water intake [8–10, 20]; low-volume, highly-concentrated urine output [7, 22, 23]; and expression of the hormone AVP [29–31]. Acting upon V2 receptors expressed in the kidney, AVP appears to contribute to the progression of chronic kidney disease and decline in glomerular filtration rate [29, 30] and may be involved in the progression of autosomal dominant polycystic kidney disease (ADPKD) [32]. Moreover, this hormone is also associated with vascular function [31] and regulation of blood glucose [10, 33]. The action of AVP on V1 receptors, expressed in vascular smooth muscle cells and mediating vasoconstriction, also suggests that AVP may be involved in the regulation of blood pressure and that its chronic hyperexpression may be linked to hypertension, though more work is needed to understand its long-term effects. An observational study of normotensive and hypertensive patients [31] revealed higher plasma AVP in hypertensive subjects than in normotensive subjects and also demonstrated a linear relationship between AVP, systolic, and diastolic blood pressure. Notably, the AVP concentration reported in hypertensive subjects is consistent with the AVP concentration measured in subjects with U_Osm_ > 500 mOsm·kg^−1^.

There is insufficient evidence to define a range for “healthy” plasma AVP concentration. However, indirect evidence for the benefits of lowering plasma AVP can be seen in treatment advances in ADPKD. Tolvaptan, an AVP-V2 receptor inhibitor, has been demonstrated to be effective in slowing the rate of cyst growth and kidney function decline [34]. A logical next step that must be evaluated is whether reducing plasma AVP via increased water intake, instead of via pharmacological blockade, is equally effective. While preliminary evidence in rats suggests that water intake is indeed effective in slowing the progression of the disease [34], a threshold for a “healthy” plasma AVP concentration remains to be established.

There are limitations to the analytical approach presented above. The associations between fluid intake, U_Osm_, chronic disease, and AVP are derived mostly from cross-sectional and cohort studies. Prospective, randomized controlled studies and further work to confirm or refine this index are sorely needed. However, the strength of the present analysis is that several approaches independently point towards a similar value of U500 as an index representative of adequate intake. A major advantage is that the method can be applied to a variety of populations and situations. Urine osmolality reflects the net sum of all fluid-electrolyte regulation; therefore, it intrinsically takes into account differences in diet-related osmotic load, total daily water consumption, climate, body size, sweat loss and water gained via metabolism in those who exercise strenuously. Thus, for the individual, U500 may reflect a daily water intake that is sufficient not only to meet the physiological water requirement, but also to ensure adequate urinary excretion and downregulate AVP secretion, both of which may reduce the risk of chronic renal and metabolic disease.

## 5. Conclusion

Excreting a low volume of concentrated urine appears to have costs that are both direct (i.e., faster decline in GFR and incidence of kidney stone recurrence) and indirect (i.e., association between increased circulating AVP and glycemic control, diabetes, hypertension, and ADPKD). Thus, maintaining dilute urine may have some benefits; however, today, the precise dose-response relationship between water intake, U_Osm_, and disease risk is not sufficiently clear. Growing evidence suggests that a daily fluid intake above that which is physiologically necessary for water balance is desirable. Maintaining a sufficiently low 24 h U_Osm_ (i.e., via increased total daily fluid intake) reduces the antidiuretic effort of the kidneys and is therefore an easy and cost-effective way to reduce the negative effects observed in association with increased AVP concentration. Maintaining a 24 h U_Osm_ below 500 mOsm·kg^−1^ may thus be considered as a simple index of optimal hydration.

## Figures and Tables

**Table 1:** Bivariate relationships between total fluid intake (TFI), 24 h urine volume (U_vol_), and osmolality (24 h U_Osm_).

Reference	Description	Criterion value	U_Osm _cutoff value derived from ROC analyses	Sensitivity	Specificity
EFSA [1]	Dietary reference value for total fluid intake (estimated to be 80% of total water intake)	TFI ≥ 2.0 L·d^−1^ (men) or ≥ 1.6 L·d^−1^ (women)	544 mOsm·kg^−1^	0.86	0.80

Curhan et al. [4]	Multivariate-adjusted odds-ratio (OR) for kidney stones in women with higher TFI	TFI ≥ 1850 mL·d^−1^	525 mOsm·kg^−1^	0.95	0.77

Borghi et al. [23]	In recurrent stone formers, increasing 24 h U_Vol _resulted in a 50% lower stone recurrence rate over 5 years	24 h U_Vol_ ≥ 2.0 L·d^−1^	448 mOsm·kg^−1^	0.98	0.82
